# On the Track of DNA Methylation: An Interview with Adrian Bird

**DOI:** 10.1371/journal.pgen.1000667

**Published:** 2009-10-16

**Authors:** Jane Gitschier

**Affiliations:** Departments of Medicine and Pediatrics, Institute for Human Genetics, University of California San Francisco, San Francisco, California, United States of America

The day school let out for the summer, my daughter and I packed our bags for Britain, where we had lived for a few months in 2006. Annie was eager to reconnect with her friends there, and I had arranged to conduct three interviews. In desperation and with the clock ticking, I struggled to fit my bulky recorder into my wheelie when it dawned on me that the “talk app” on my daughter's iphone should be up to the job. You can imagine the reluctance and skepticism on the part of my 15-year-old, but she managed to get into the spirit and acquiesced.

First up on my schedule was Adrian Bird (Image 1), who holds the Buchanan Chair of Genetics at the University of Edinburgh and is also Director of the Wellcome Trust Centre for Cell Biology. Long before the word “epigenome” was coined, Bird began mapping the distribution of DNA methylation (occurring at the cytosine of CpG dinucleotides) in the genomes of a variety of species. His work emerged just as agarose gels, restriction enzymes, and Southern blots were being developed. Bird later spawned the idea of CpG islands, pockets of DNA rich in unmethylated CpGs and frequently found in conjunction with the promoter regions of mammalian genes. Bird's observation provided a roadmap for disease gene discovery for about 15 years, until human genome draft sequences began to emerge.

**Figure:**
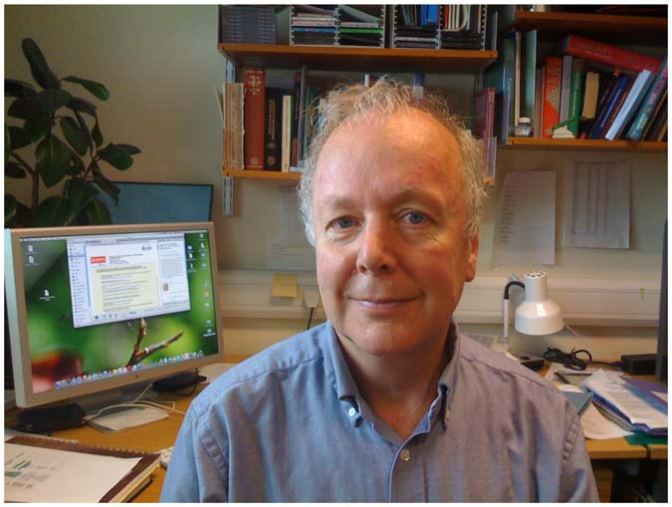
Adrian Bird.

Bird's laboratory then went on to identify proteins that bound to methylated DNA, one of which (MeCP2) was discovered years later to be defective in Rett Syndrome, a rare X-linked disorder in which affected girls develop autism and a distinctive set of behaviors. This astonishing turn of events propelled Bird to extend his studies on MeCP2 to a murine model for Rett Syndrome, ushering in new ideas about therapy for this devastating illness, but still leaving open the question of MeCP2's role in the brain.

Bird and his wife Cathy Abbott, also a geneticist, invited me to spend the night prior to the interview with them (future interviewees, take note!), and I was delighted to do so. Still jet-lagged, I traveled by train, leaving behind the uncharacteristic sun of Cambridge to find cold rain penetrating the skylights at Edinburgh's Waverley Station. It felt very cozy to share the evening with them and their children, Tom and Annie: chatting, watching some Twenty20 (an abbreviated form of cricket), playing Uno, feeding the three guinea pigs, and experimenting with the iphone's tape app, which, to our delight, worked.

**Gitschier:** My first question is a two-part, integrated one. How did you get interested in methylation, and what was the state of the art at the time you started working on it?

**Bird:** I first got interested when I was in Zurich doing a post-doc.

**Gitschier:** Whom were you with there?

**Bird:** Max Birnstiel. I had been in the States doing a post-doc [with Joe Gall at Yale] on gene amplification in frog oocytes. When I went to Max's in Zurich, he had a visitor named Ham Smith.

**Gitschier:** What year approximately was this?

**Bird:** 1973–1974. Ham was on sabbatical, and the first thing he did was to make a restriction enzyme, HpaII—*Haemophilus parainfluenzae* II.

I was making ribosomal RNA genes, just for something to do really. We knew there was a difference between the amplified ribosomal RNA genes, which were extrachromosomal in the oocyte, and the chromosomal ones, and that the difference was due to methylation. Don Brown and Igor Dawid had shown that chromosomal rDNA had 5-methylcytosine and the amplified didn't.

So I used HpaII to cut this amplified [extrachromosomal] DNA, and it cut beautifully. But when I tried with purified chromosomal ribosomal DNA, it didn't cut. It was known that restriction enzymes were blocked by methylation in the organisms from which they are derived; there is usually a restriction enzyme and a modification enzyme that matches it and that protects the genomic DNA of the host from its own destructive enzymes. So it seemed that some of the methylation [in the chromosomal rDNA] might be mimicking the blockage that occurs in the *Haemophilus parainfluenzae* endogenous enzyme.

**Gitschier:** Did Ham Smith know that HpaII didn't cut methylated DNA before you did the experiment?

**Bird:** Probably, but he just made restriction enzymes in order to make himself at home. Restriction enzyme technology was new, and agarose gels had just been brought in. And another guy there was Ed Southern, who had just invented Southern blotting, so there was a bit of coincidence here.

**Gitschier:** Was Ed on sabbatical?

**Bird:** Yes, he was as well. I can't remember whether they were there at exactly the same time. I think they overlapped.

**Gitschier:** So, why is everybody coming to Max Birnstiel's lab?

**Bird:** Max Birnstiel and Don Brown were hotly competing groups because both of them worked on ribosomal RNA genes, which you could purify by buoyant density centrifugation. Before you could clone DNA, the only way to get hold of pure gene was A) because it was highly repeated and B) because its buoyant density was different from the bulk [of the DNA]. So if you ran enough cesium chloride gradients you could get it pure and then you could study the structure of it. Frog ribosomal genes and sea urchin histone genes were where it was AT in those days.

**Gitschier:** What attracted Ham to that lab?

**Bird:** Max and Ham knew each other and were friends.

**Gitschier:** So for him, it was going to a friendly and interesting place. And Ed?

**Bird:** He came over to work with Max, I have to say, partly because he was going out with someone who happened to work in Max's lab. In those days, people's motives were more random than they might be made to look now.

**Gitschier:** Sounds lovely. So Ham's making this enzyme, and you are just trying this enzyme, you don't know what the results are going to be.

**Bird:** I'm not sure what I'm doing, to be honest! I knew I wanted to do something interesting, but I was just playing around more than anything else. Ham was playing around with the enzymes he knew about and Ed was doing the technological things that he really liked doing. So accidentally this gave rise to the idea that one could use restriction enzymes to map methylation in DNA. There was a conjunction of areas that were needed before one could exploit this properly, and that didn't happen until I got back to Edinburgh.

By 1975, I was back there in an MRC unit and mapping the methylated and nonmethylated sites in the ribosomal genes in *Xenopus laevis*. And it took absolutely ages to get that published.

**Gitschier:** At some point you moved away from frog oocytes.

**Bird:** We looked at sea urchins—invertebrates—and found there was both a methylated and an unmethylated fraction of the genome. The last things we came to were vertebrate cells and they didn't seem to have anything like what you see in the invertebrates. When you digested the DNA with these methylation-sensitive enzymes, nothing happened because most of the DNA is methylated.

Then we had the idea that maybe we could see a small fraction of unmethylated DNA if we end-labeled it. That was the work of David Cooper who did a Ph.D. in my lab. I can remember him doing the first end-labeling, because it made a horrible blob. Something that could be artifactual, but it wasn't, and we spent quite a lot of time showing that. I cloned mouse fragments derived from that blob, and that was our 1985 paper in *Cell*. And then we restriction-mapped them and showed that they came from clusters of nonmethylated CpGs in the genome.

We were not totally alone in reaching these conclusions. Tykocinski and Max had looked at DNA sequence in MHC class I genes and saw these clusters, and earlier a guy called De Crombrugghe also saw something like this. There was also stuff on the inactive X chromosome—Barbara Migeon had seen nonmethylated sequences at the 5′ ends of genes.

It was in the air, but not yet an accepted generalization. Our data really suggested that there was a category of genomic DNA that was full of CpGs that weren't methylated and that eventually were understood to be near promoters. So we kind of brought it all together.

**Gitschier:** So you published this review article [in *Nature*], which is where I became familiar with your work.

**Bird:** Yes, that was cited loads of times, because it was the mapping phase of the genome project where people wanted to map themselves into reality.

**Gitschier:** Well, people used them to find genes. They were like little flags that said, “Hey over here, I'm a gene!”

**Bird:** Exactly.

**Gitschier:** Did you coin the name “CpG islands”?

**Bird:** No I didn't. We called them “HTF islands” for “HpaII tiny fragments”, but reviewers said, “What the hell is HTF?” It was Marianne Frommer, who was doing a sabbatical in my lab at the time the *Cell* paper was published, who called them CpG islands—it made more sense.

**Gitschier:** OK, let's switch gears. At some point, you started to work on proteins that bound to methylated regions.

**Bird:** That arose by chance as well. The CpG islands provide an approximate way to map methylation throughout the genome, but at the time there was no way to do that properly. So we kind of ran into a brick wall—what you really want to know is where the methyl groups are throughout the genome.

**Gitschier:** And you found out where they weren't.

**Bird:** So I decided to work on how the CpG islands might originate. We asked, “Does something bind to the nonmethylated sequence that might protect it from methylation?” Just by steric inhibition. We made an oligonucleotide, and at the time it took Amersham about 4 months to do it. I just made up a sequence full of CpGs that were in restriction enzyme sites so we could test their methylation status, and then we oligomerized them and methylated the sites using commercial enzymes, because you could buy HhaI and HpaII methyltransferases.

Then we made extracts from mouse liver nuclei. And what we found was something that bound to the methylated one but not the unmethylated one. So after a period of time where we considered whether this was interesting or not, we decided to work on isolating what bound to the methylated DNA.

**Gitschier:** What year are we now?

**Bird:** This is 1984–1985.

**Gitschier:** So, if you already knew about this protein you called MeCP1, why did you go on to look for more methylated-DNA binding proteins?

**Bird:** We were trying to purify MeCP1, and we were mucking about changing the assay, and in doing that we detected another protein. We couldn't purify MeCP1 for love nor money, but it has been purified subsequently by Yi Zhang. However, we could purify a different protein, which we called MeCP2. It was relatively easy.

**Gitschier:** What, then, did you do with……MePC2 [sic]?

**Bird:** Yeah—terrible name. Entirely my fault. Like HTF islands. I have a talent for inventing awkward names.

First thing we did was dissect out the methyl-CpG binding domain. These were not high-profile publications, because no one was interested in MeCP2 for a long time. Then we wanted to know if it was a methyl-binding domain in vivo, as opposed to in vitro. And that is STILL an issue that people are not convinced about. But I am.

We showed that it went to the satellite foci in mouse nuclei. In mouse, the pericentromeric heterochromatin is quite CpG rich and heavily methylated. MeCP2 goes there and stains in “spots”. It's about 10% of the genome, but contains half of all the methylation. It was a nice visual way of showing binding. Then we used a DNA methyltransferase mutant that has much lower levels of methylation, and we no longer had staining of the heterochromatin. So that said that it was the methylation that was causing it to bind. That was quite an important paper because it showed that this mindless, in vitro assay that we used for purification actually had some biological relevance.

Next we decided we wanted to know what MeCP2 did, so we knocked it out. And then Zoghbi and Francke showed that it was mutated in Rett Syndrome.

**Gitschier:** I didn't appreciate that. You actually did make a knockout before they found it as the basis for Rett?

**Bird:** Yes. The reason you didn't appreciate it was because we didn't get the right result. In something like 1993, we knocked it out. Because it's on the X chromosome, you've got a null immediately in male ES cells. We made chimeras and the chimeras all died. We concluded that it was an embryonic lethal.

By the time the Rett Syndrome story came out, we had already decided to do it again and made a conditional knockout. I now think that growing ES cells in the absence of MeCP2 somehow compromises their ability to form embryos properly.

**Gitschier:** So you didn't get a result that was really wrong, you just didn't get a result that was really useful in studying Rett Syndrome.

**Bird:** Correct. We actually started on this conditional MeCP2 knockout before the Rett Syndrome story came out. We knocked MeCP2 out in early embryonic development. Those [male] mice were born and normal until about 6 weeks and died at about 12 weeks. And then we did it again just knocking out in neurons and glia with the same result. This said the phenotype was entirely due to the brain.

**Gitschier:** OK, so you're working away on trying to understand the function in mice, and suddenly Zoghbi and Francke labs discover that the gene you've been working on has real human consequence.

**Bird:** Yeah.

**Gitschier:** Were you a reviewer on that paper?

**Bird:** Yes.

**Gitschier:** What did you think when you saw the paper?

**Bird:** I thought, “What the hell is Rett Syndrome?”

**Gitschier:** I was going to ask you that!

**Bird:** In my collection I did have papers about it, but I hadn't been keeping up with it at all, honestly.

**Gitschier:** What else did you think?

**Bird:** I thought, well we're already working on these mice, so now let's see if we can make a model for Rett Syndrome as well.

**Gitschier:** But what about the physiological or mechanistic implications? The fact that defects in a methyl-CpG binding protein cause a neurological disorder seems wild to me.

**Bird:** We're working on that quite a lot at the moment.

I think the people who were looking in the interval for the gene that was mutated were quite disappointed to find that it wasn't one of the GABA receptors, which is in that region, but rather that it was this rinky-dink little housekeeping gene. No one could get terribly inspired by finding it. But to me, it was great.

It is quite exhilarating to realize you have some preliminary insights into something whose function turns out to be very important. I had previously been rather disparaging about medically relevant research, considering that I was working on pure knowledge and that biological information was intrinsically important. But as soon as you collide with biomedical relevance, it changes your perspective. It breathes life into the project and it has added new dimensions to my research. I'm absolutely delighted it happened.

But even now, not everybody agrees about what MeCP2 does.

**Gitschier:** Let's turn to that now, because to me that is very murky.

**Bird:** The initial experiments said [the following]: A) It's a methylated-DNA binding protein. B) It's very abundant in brain. In fact, that's where we purified it from. C) It's associated with a co-repressor called Sin3a and is able to repress transcription in model systems by co-transfection in cultured cells. So the prevailing view is that it's a methyl-CpG binding repressor.

But, if you look in the knockout you find a lot of genes go up and a lot of genes go down, whereas you'd expect, if it's a repressor, that genes would only go up. So this has made everyone think twice.

But another argument, which people don't usually find persuasive I have to say, is that some genes going up and some genes going down makes sense if MeCP2 is regulating very many genes. Because you've probably got a closed system—for everything that goes up there must be something that goes down. A good example of this—if you treat cells with the histone deacetylase inhibitor TSA (trichostatin A), this causes histone acetylation to go through the roof, which is a marker of activity, but expression-wise as many genes go up as go down. Everything would LIKE to go up, but you can't employ more polymerases, etc., than are there.

We've been through phases where we have been prepared to throw out all the old stuff and say neurons are doing something different with MeCP2. But the data have forced us to come back to the original idea that MeCP2 coats the genome and recruits enzymes that deacetylate and keep the acetylation low.

So what is its function? Mike Greenberg [Harvard Children's Hospital] showed that MeCP2 is a phosphoprotein. It gets phosphorylated when neurons are active. We know that when neurons fire, you get bursts of synaptic protein synthesis and nuclear protein synthesis and this is associated with plasticity. It is an attractive idea that MeCP2 has something to do with that. Neurons fire—phosphorylation of MeCP2 changes its properties—let's say it comes off, leading to a burst of transcriptional activity, which is somehow involved in neuronal homeostasis. It's an intriguing possibility.

To be honest, the reason that there are so many theories about what MeCP2 does is that none of them has been nailed down experimentally. There is a bit of a vacuum there, and people fill vacuums with speculations, as they should. All of the things I'm saying are subject to different views from different people, but I would say MeCP2 is involved in maintenance.

**Gitschier:** When you say maintenance—do you mean maintaining the neuron itself, or maintaining the neural activity?

**Bird:** Functional integrity. What seems to degrade in Rett Syndrome is neuronal activity. The neurons aren't dead. What you'd like to say is that neurons are degenerate, but you're not allowed to say that, because neurodegenerative disorders, like Alzheimer or Parkinson, involve neuronal death.

**Gitschier:** We think of autism as a neurodevelopmental defect and Rett as being in that category as well, because its onset is during the development of a human being.

**Bird:** The reason that people are so wedded to the idea of Rett being neurodevelopmental is that of course there are lots of things going on in neurodevelopment at the time girls get the symptoms. What impresses me is that female mice get equivalent symptoms between 4 and 12 months and humans between 6 and 18 months—in REAL time, that is the same, more or less. But developmentally, they are at totally different stages.

People see only these two categories [neurodegenerative and neurodevelopmental], but I would say it's a neuromaintenance disorder. The best evidence for that is reversibility [see below].

Neurons are long-lived complex cells that expend a lot of effort deciding who they are going to be connected to. As a neuron you'd better remember all that because lots of things are going to happen to this organism over a period of years, or decades in our case, and that neuron will not get a chance to renew. So maintenance becomes an extremely important problem, and I think MeCP2 is one of the proteins that have evolved to ensure that.

In my opinion the reversibility experiments that we did are interesting for all sorts of disorders in which neuronal death has not been established, and that includes autism and schizophrenia, for example. The general assumption is that once you've got one of these neurological disorders, that's it. When one sees Down's Syndrome, or mental retardation of any kind, it is ingrained in us to believe that nothing can ever be done to reverse that.

**Gitschier:** Tell me about the reversibility experiments.

**Bird:** That's probably one of the most impressive things we've ever done. I'll show you a movie. Neurons don't die in Rett Syndrome or in the mouse model. So this raised the possibility that one could put the gene back and find out whether the symptoms are reversed. Jacky Guy took MeCP2 and inserted a stop cassette in an intron—designed to prevent expression, but flanked by loxP sites. When you mate that with a mouse with Cre expressed under estrogen receptor control, you can inject tamoxifen, release Cre, delete the stop, and presto transcription starts again. It was one of those projects where everything worked even though we thought it might never work.

Here's the life cycle of a male MeCP2 stop-cassette mouse. It is born and then gets symptoms at 6–8 weeks and dies at 9–16 weeks. We inject MeCP2 here, when the mouse has advanced symptoms and is near death. It doesn't really move, very low to the ground, feet splayed apart, tremor, arrhythmic breathing. Now, here is the same mouse 4 weeks later.

**Gitschier:** Oh my goodness. It looks exactly like the control.

**Bird:** We've done this with many mice and it's consistently reversible. Females breed normally for about 6 months, but after that they hit a brick wall, become inert and obese, develop hind limb clasping, tremors, and arrhythmic breathing—all the things that mimic aspects of Rett Syndrome, and it is stable, just as in humans, and lasts for the rest of their lives. That is the real model for Rett Syndrome. These animals are way beyond neurodevelopment. But even at this late stage, it is reversible. It establishes the principle that Rett Syndrome, as least in mice, is reversible, and it encourages the belief that it might be reversible in humans, too.

I must say the reversal is the most surprising result we've ever had, partly because it went completely against what was expected and partly because I'm a biochemist at heart and this was a sophisticated genetic problem. It was nice to follow the problem into the mouse and do an experiment that has had an impact beyond Rett Syndrome and that many neuroscientists find interesting. I'm now on the fringes of that world, and I'd like to try and make more contributions there.

**Gitschier:** So you're not about to stop soon, I take it!

**Bird:** Everybody knows that you yourself are probably not the best person to judge that, but I don't feel in the slightest like stopping.

When people write and say that the mouse reversal has transformed their view of prospects for their daughter, you are flattered by that, but it very quickly gives way to frustration—that you've raised people's hopes but you cannot in any way replicate the reversal in humans. Although it seems like a short step to a lay person, in fact, it is a gigantic leap into the unknown.

